# Detection of human parvovirus 4 DNA in the patients with acute encephalitis syndrome during seasonal outbreaks of the disease in Gorakhpur, India

**DOI:** 10.1080/22221751.2018.1563455

**Published:** 2019-01-21

**Authors:** Vidya A. Arankalle, Navin Srivastava, Komal P. Kushwaha, Agnibha Sen, Ashwini Y. Ramdasi, Priyanka A. Patel, Sumeet Kuthe, Bangari Haldipur, Gajanan N Sakpal, Kavita S. Lole, Nilesh B Ingle

**Affiliations:** a National Institute of Virology, Microbial Containment Complex, Pune, India; b Interactive Research School for Health Affairs, Pune, India; c Baba Raghav Das Medical College, Gorakhpur, India

**Keywords:** NGS, HPARV4, phylogenetic analysis, acute encephalitis syndrome, Gorakhpur, *Orientia tsutsugamushi*

## Abstract

Seasonal outbreaks of acute encephalitis syndrome (AES) at Gorakhpur, India have been recognized since 2006. So far, the causative agent has not been identified. Use of next generation sequencing identified human parvovirus 4 (HPARV4) sequences in a CSF/plasma pool. These sequences showed highest identity with sequences earlier identified in similar patients from south India. Real-time PCR detected HPARV4 DNA in 20/78 (25.6%) CSF and 6/31 (19.3%) plasma of AES patients. Phylogenetic analysis classified three almost complete genomes and 24 partial NS1 sequences as genotype 2A. The observed association of HPARV4 with AES needs further evaluation. ELISAs for the detection of IgM and IgG antibodies against scrub typhus (*Orientia tsutsugamushi*, OT) showed ∼70% IgM/IgG positivity suggestive of etiologic association. Prospective, comprehensive studies are needed to confirm association of these agents, singly or in combination with AES in Gorakhpur region.

## Introduction

Outbreaks of Japanese encephalitis (JE) have been reported from the Gorakhpur district and adjoining areas of Uttar Pradesh state, India, during 1978–2005 [1] leading to the introduction of JE vaccine [2]. Following vaccination, JE accounted for <10% of AES cases admitted to the BRD Medical College, the only tertiary care hospital catering to the AES cases in Gorakhpur division [2]. During 2007–2014, variable high proportion of AES cases with unknown etiology (41.6–61%) were recorded [3–5]. Over 2000 AES patients are admitted annually to this hospital with high case fatality ratio (20–30%) [6]. With the dramatic rise in non-JE cases, vigorous attempts were made by the National Institute of virology, Pune, India to identify the agent by in-vivo (suckling mice) or in-vitro (several cell-lines, PCR) amplification without any success (unpublished observations). In view of the failure of classical and molecular techniques in identifying the agent, in 2011, we decided to employ Next Generation sequencing (NGS) technology not requiring prior sequence information to explore the possibility of detection of the agent(s) in the clinical specimens. At the time of this study, enteroviruses were reported in 21.3% of the patients [7]. Subsequently, association/role of scrub typhus (*Orientia tsutsugamushi*, OT) in causing AES outbreaks was extensively studied [8–11]. We present our NGS data and reanalyse the findings in relation to the current perspective.

## Results

### Patient characteristics

For HPARV4, we investigated 78 paediatric patients (64 in 2011 and 14 in 2012) from whom a CSF sample was available (Table 1). These included 46 (58.9%) children below the age of five, 21 children (26.9%) in 5–10 years while 11 were of 11–15 years age (14.1%). All the patients came with fever of 2–13 days (8.3 ± 0.27 days). Most of the patients were undernourished, 31% were below −3 SD weight for the ages and 66% were between −2SD and −3SD (WHO growth standards 2006). Table 1 presents the most common presenting symptoms. In addition, 10% children had non-palpable dark, irregular macular rashes on the trunk. ECG abnormalities like low voltage, QRS complexes, tachycardia and abnormal elevation of ST segment and inversion of T-waves were recorded in 10% children. Radiological evidence of cardiomegaly was observed in 14% children. The CSF was clear and 50% of them had pleocytosis and raised CSF protein (40–100 mg/dL in 62 children); CSF sugar was normal or more than 60% of the blood sugar; 60% had leucocytosis, mainly Polymorphonuclear leucocytosis. Additional patients investigated for OT antibodies were similar except that the samples were collected during 1–7 days post-onset of clinical symptoms (3.7 ± 0.32 days).

**Table 1.** Proportion of 78 AES cases exhibiting different symptoms.

Type of symptoms	Percent children exhibiting symptoms
Loss of interest in play	75
Feeling/appearing sick	60
Unconsciousness	41
Vomiting	41
Upward rolling of eye ball	41
Convulsions (Focal seizures)	79
Swelling over face & limbs	34
Muffled heart sound	34
Tachycardia disproportionate to fever	31
Pallor	31
Respiratory distress	21
Abdominal distension	14
Headache	10
Body ache	31
Constipation.	3
Hepatomegaly	69
Spleenomegaly	21
Extensor planter reflex	97
Hypertonia	14
Hypotonia	6
Lymphadenopathy	3
Papilloedema	3
Hypotension	14

### Identification of viral RNA/DNA sequences employing NGS platform

As the use of total nucleic acids for the detection of RNA or DNA viruses yielded unsatisfactory results for both categories, different samples were used for RNA and DNA detection (Table 2). The samples exhibited presence of a large number of bacterial, fungal contigs that were also detected in control samples and hence were not investigated further (Supplementary Table 1). The detection of Tobacco Mosaic virus was surprising (Table 2). We did not detect enterovirus sequences.

**Table 2.** Samples used for the detection of the agent (1–12 for RNA and 13–22* for DNA).

_Sample ID_	_Age/sex_	_Sample types_	CLC		VELVET	
			Viral /total contigs	Virus	Viral /total contigs	Virus
GKP-1-a	12F	Brain	0/46		0/594	
GKP-1-b	12F	Kidney/Heart/Liver	0/296			
GKP-2-a	3M	Liver	0/18		0/736	
GKP-2-b	3M	Heart	0/12		0/476	
GKP-2-c	3M	Brain	0/48		0/7778	
GKP-2-d	3M	Kidney	0/25		0/948	
GKP-3	1.5M	CSF/Serum	1/76	ReMV^1^	0 l/205	
GKP-6	1.5M	PBMC	0/184	–	0/354	–
GKP-7	7F	Plasma	10/257	TMV	22/6015	21TMV, 1BVDV^4^
GKP-8	6M	Plasma	2/137	TMV	2/260	TMV
GKP-9	7F	Plasma	0/118		0/434	
GKP-10	11F	Plasma	0/218		0/336	
GKP-11	8F	Plasma	0/184		1/869	ReMV
GKP-12	5F	CSF	0/18		0/45	
GKP-13	3F, 7F, 6M	1 CSF + 2 sera	2/54	PARV4^5^	5/56	PARV4
GKP-14	3F	Liver/kidney/heart	1/151	B19^6^	5/160	4B19, 1PARV4

*Two sets of barcoded samples of 4 CSF each (15–18, 19–22) yielded less data.

1 = Rehmania Mosaic virus, 2 = Tobacco Mosaic virus, 3 = Cucumber Mosaic virus, 4 = Bovine viral diarrhoea virus, 5 = Human Parvovirus 4, 6 = Parvovirus B19.

### Detection of Human Parvovirus 4 DNA

The first run employing CSF/serum pools identified 12 contigs of Human Parvovirus 4 when different tools were used for analysis (Tables 2 and 3). These were closely related to the sequences reported from CSF of two AES patients from south India [12]. Further, when 45 reads ≥ 100 bp were mapped with the reference PARV4 genome (Accession No HQ593530, 5205 nt), almost complete genome was covered with 8 gaps of 17/19/46/65/217/289/316/338 nt. In the pooled tissue sample (GKP-14), five contigs of Human parvovirus B19 and one contig of PARV4 were identified. Use of two barcoded CSF samples of four each did not yield any data, probably because of lower DNA/sample.

**Table 3.** Details of PARV4 DNA sequences identified employing different tools.

Analysis type	GKP-13		GKP-14	
	Contigs No	Length (bp)	Contigs No	Length (bp)
CLC (human filter)	2	400, 276	–	–
CLC (No Human filter)	5	278, 357, 422, 508, 1050	–	–
Velvet (Human filter)	4	210–249	1	240
No. of raw read ≥ 100 bp mapped on viral genome (% genome coverage)	45 (74.2%)		55 (32.7%)	

Note: NA: not applicable.

### HPARV4 DNA positivity and phylogenetic analysis

**Figure 1.**
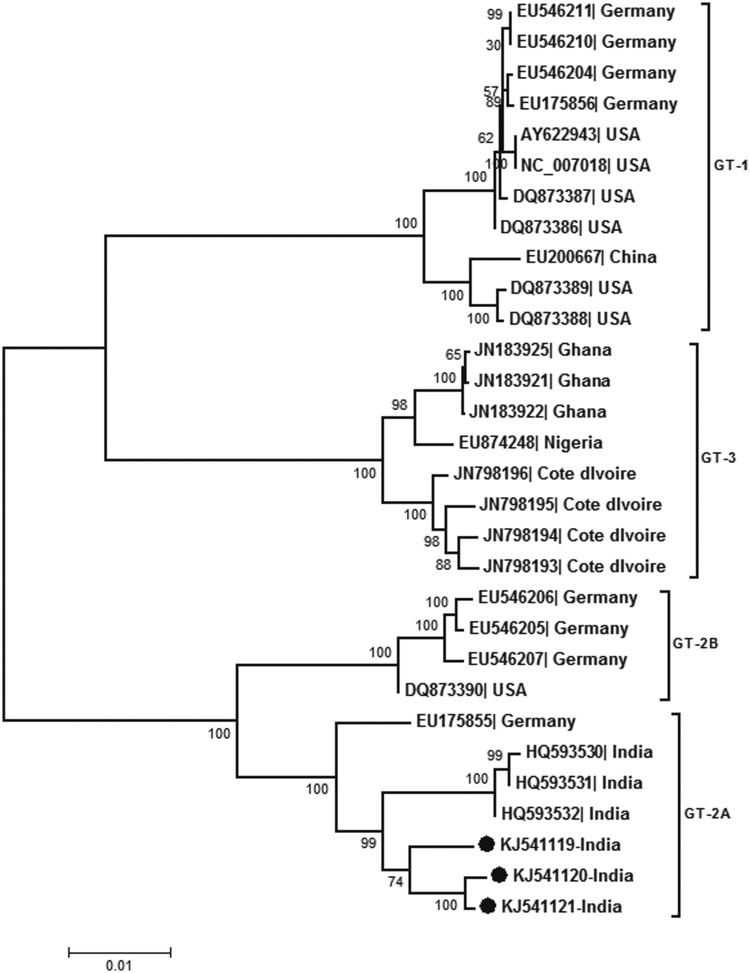
Phylogenetic analysis of almost full genomes sequenced during this study (accession numbers KJ541119, KJ541120 and KJ541121). Full genome sequences available in the Genbank database are denoted by the respective accession numbers. Percent bootstrap support is indicated at each node. Genotypes are designated as brackets. Solid circles denote sequences obtained during the present study. Scale bar indicates nucleotide substitutions/site.

**Figure 2.**
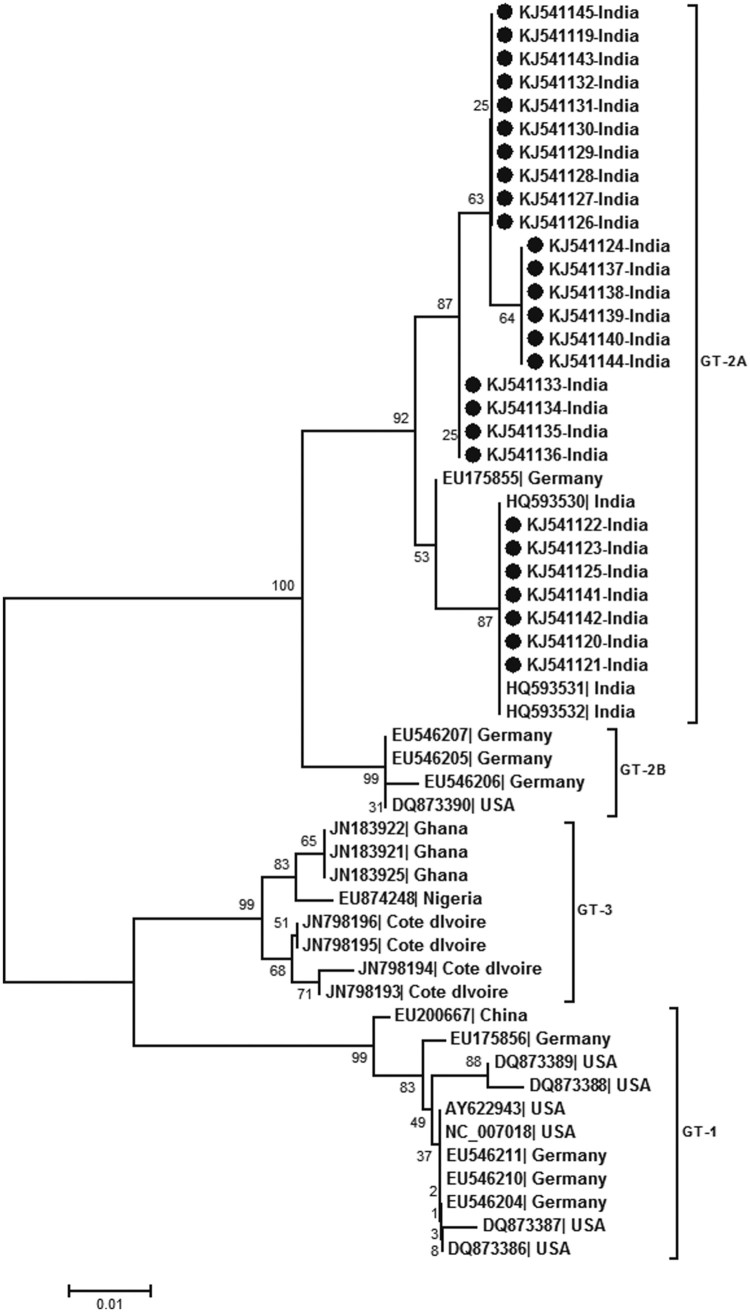
Phylogenetic analysis of partial NS1 region (256 bp) sequenced during this study (accession numbers KJ541119 to KJ541145). The other sequences used for analysis are denoted by the respective genbank accession numbers. Percent bootstrap support is indicated at each node. Genotypes are designated as brackets. Solid circles reperesent sequences obtained during the present study. Scale bar indicates nucleotide substitutions/site.

Real-time PCR (detection limit 10 copies/reaction) detected HPARV4-DNA in 20/78 (25.6%) CSF and 6/31 (19.3%) serum samples (10^2^–10^10^ copies/ml). HPARV4 positivity was comparable during both the years, though the sample size in 2012 was small (18/64, 28.1% and 2/14, 14.3% respectively). HPARV4 DNA was not detected in the serum samples from the healthy controls from Gorakhpur as well as Pune. Almost full genome sequences were generated for three positives with high viral load (accession numbers KJ541119–21) while partial NS1 was sequenced for additional 24 positives (18 CSF, 6 sera; accession numbers KJ541122-KJ541145). Phylogenetic analysis (Figure 1) classified all the sequences as genotype 2. Within genotype 2, the Indian sequences from Gorakhpur (this study) and south India [12] formed distinct clusters, the percent nucleotide identity being 98.1 + 0.005%. Genotype 2 was further subdivided into two groups with a percent nucleotide difference of 3.8 ± 0.005%. The intra-genotypic difference was 7.4–8.9%. The phylogenetic pattern employing partial NS1 region was identical to full genome-based analysis (Figure 2).

### Testing for IgM and IgG antibodies against *Orientia tsutsugamushi*

**Figure 3.**
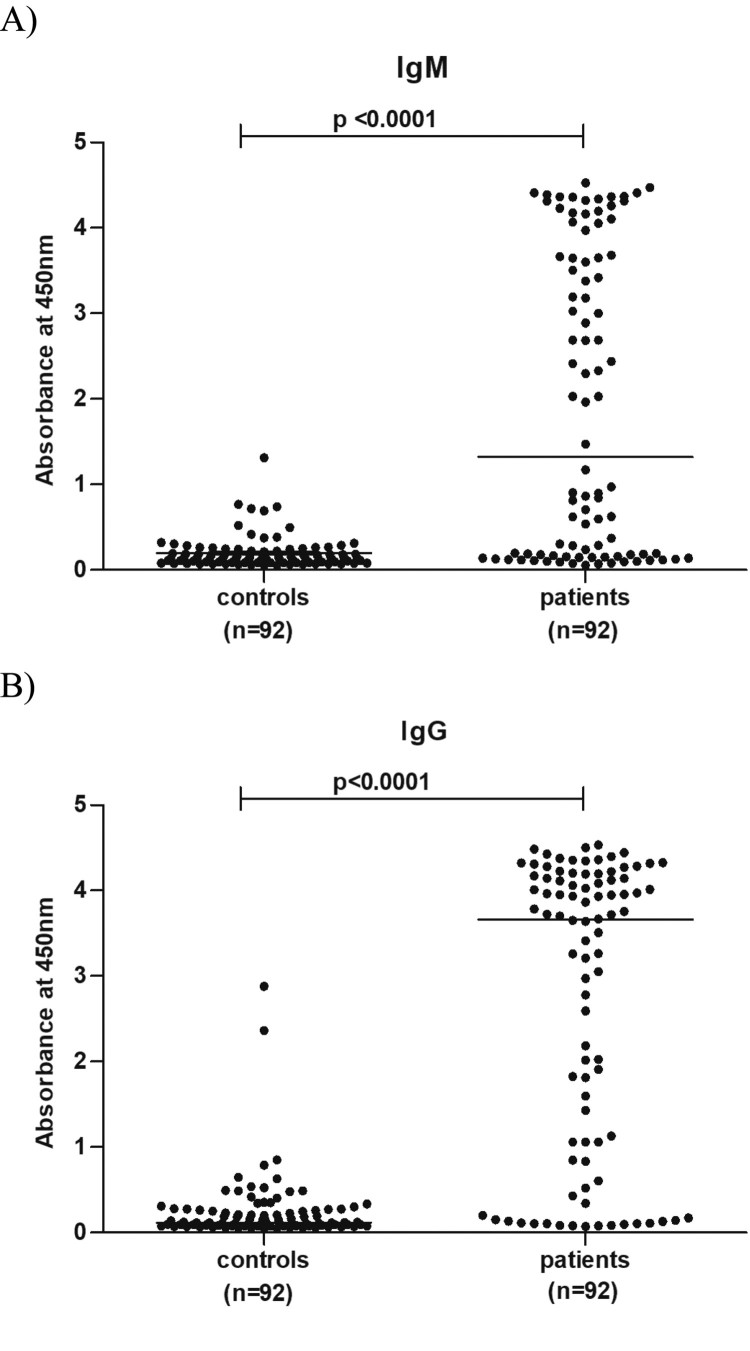
Optical density values for 92 samples each from patients and controls in ELISAs done for the detection of IgM and IgG-anti-OT antibodies.

In view of the high positivity of these antibodies among patients investigated during the subsequent years [8–11], we tested available serum samples for both the antibodies (Table 4). These included 80 patients from 2011 and 12 patients from 2012. High IgM positivity in patient category than in controls was striking (*p* < .0001). As depicted in Figure 3, the patient population was characterized by high OD values for both the markers, while 88–95% of the control children population was susceptible to OT as evidenced by the absence of IgG antibodies. Of the 31 serum samples tested earlier for HPARV4, 14 could be subjected to ELISA testing for OT antibodies. These included two HPARV4 DNA positives. One each was positive for IgM/IgG and IgG antibodies.

**Table 4.** IgM and IgG-anti OT positivity in AES and control children.

ELISA reactivity pattern	No positive/no tested (%)		
	Patients (1.6–10 years)	Controls (<5 years)	Controls (>5–10 years)
IgM+ IgG+	54/92 (58.7)	0/42	0/50
IgM+ IgG-	4/92 (4.3)	1/42 (2.4)	2/50 (4)
IgM- IgG+	18/92 (19.5)	1/42 (2.4)	4/50 (8)
IgM- IgG-	16/92 (17.4)	40/42 (95.2)	44/50 (88)

## Discussion

Our data reconfirms the use of NGS platforms in the identification of unknown agents. We tried both RNA and DNA isolation-based detection of the suspected agent. However, RNA-based detection could not identify sequences of a possible agent. On the contrary, blast analysis of the contigs generated by the first DNA run identified HPARV4 sequences that were closest to the earlier sequences generated from CSF samples of paediatric encephalitis patients with unknown aetiology from south India [12]. These results encouraged us to evaluate HPARV4 as the possible agent and further NGS runs with additional samples were not carried out.

We used real-time PCR [13] with sensitivity of 10 copies/reaction for the detection of viral DNA and could detect the virus in substantial proportion of the CSF samples screened. Importantly, very high viral loads were also detected in a few CSF samples (10^7^–10^9^ copies/ml). None of the 25 healthy controls were circulating HPARV4 DNA suggesting low prevalence of this virus in the general population. Of note, HPARV4 from Gorakhpur and south India belong to genotype 2 and form a distinct cluster (Figure 1), strains from US and Germany (except one) form a separate cluster whereas all the African strains belong to genotype 3 [14]. Considering intra-genotypic nucleotide difference of 7.4–8.9%, the difference of 3.8 ± 0.005% between two branches of genotype 2 may be considered as distinct sub-genotypes. It would be worthwhile to explore if genotypes influence epidemiology and/or outcome of PARV4 infection.

Human parvovirus 4 of the family Parvoviridae was discovered in a plasma sample of an adult HIV positive patient from US with an undiagnosed acute infection in 2005 [15]. The epidemiology and disease potential of PARV4 is largely unknown. As against the parenteral route of transmission in the US and certain European countries [15,16–18], and Taiwan [19], non-parenteral transmission routes were shown in African countries [20–23]. The patients investigated during this study were not at high-risk of parenteral transmission suggesting alternate transmission mechanism(s). Epidemiology of HPARV4 in India is not understood and needs a special attention. Detection of HPARV4 in the CSF samples of AES cases from southern India [12] and Gorakhpur (northern India, this study) with 98.1 + 0.005% nucleotide identity suggests association of this virus with encephalitis in Indian children. Additional studies including serology are needed to assess the contribution, if any, of this virus in causing encephalitis.

In view of a recent report [10] of etiologic association of *Orientia tsutsugamushi* with AES in Gorakhpur, we reanalysed the NGS sequence data. One contig each were detected in two RNA runs (Rickettsia *tsutsugamushi*, strain Kawasaki, gene for 16s RNA, length 373 and 241nt). However, the presence of just two contigs was not enough for us to identify this pathogen as the possible etiologic agent. Interestingly, OT serology led to striking findings. The kit used for OT serology does not provide cut off value and asks the customers to determine the same on the basis of initial testing of the normal population and confirmed OT cases. In the absence of serum samples from confirmed OT cases, we tested 92 apparently healthy children sampled during non-AES period and in the same age group for OT antibodies (Table 4 and Figure 3). It was clear that circulation of OT was not very high in the healthy children while almost 70% of the patients exhibited IgM antibodies at high levels. Similar pattern was seen with IgG antibodies as well. Association of OT infection with AES was apparent.

We would like to point out two observations here. Firstly, though during acute phase, majority of the patients circulated high levels of IgM and IgG antibodies. Even patients bled on 1st or 2nd day after the onset of clinical symptoms showed similar pattern. Those negative for IgM antibodies (*n* = 16) also circulated high IgG levels. High IgG antibodies during acute phase were previously shown to be the secondary infections [24]. Second, one would expect increased seropositivity to OT (IgG positivity) as annual seasonal outbreaks are occurring since 2006. However, the exposure of the paediatric population to OT remains small.

In conclusion, the detection of HPARV4-DNA in the CSF samples of ∼25% of the AES patients from Gorakhpur with 98.1% nucleotide identity with sequences obtained from similar cases from south India suggests association of HPARV4 with encephalitis in Indian children. OT serology suggests association of scrub typhus. Both etiologies have their own limitations that should be clarified by prospective studies screening both serum and CSF samples for HPARV4 and OT (PCR) and IgM/IgG-anti-HPARV4/OT.

## Material and methods

This study was approved by the “Human Ethics Committees” of the National Institute of Virology, Pune and the tertiary care hospital, BRD Medical College, Gorakhpur.

### Patients and clinical specimens

An AES case was defined as a person with acute (<15 days) onset of fever and a change in mental status (including symptoms such as confusion, disorientation, coma, or inability to talk) AND/OR new onset of seizures (excluding simple febrile seizures) [25]. On admission, detailed history was obtained from the parents and blood sample was taken. Whenever possible, CSF was collected. IgM-anti-JEV negative patients (*n* = 78, 2011–2012) confirming to the criteria of AES and from whom a CSF sample was collected were investigated. For testing anti-OT antibodies, patients from whom no CSF samples were available were included as no CSF and only a small number of serum samples (*n* = 21) tested for HPARV4 were available due to small quantities and repeated use.

For HPARV4, we included two types of apparently healthy children controls (age 5–8 years): (1) from Gorakhpur (*n* = 25) and (2) from Pune, western India wherein AES outbreaks are not reported. For OT, serum samples from 92 healthy children (<5 years = 42; <5–10 years = 50) were examined. These samples were collected earlier for vaccine immunogenicity study during non-epidemic period and stored at −20°C till tested.

Twenty-two acute-phase specimens were used for NGS experiments employing Ion torrent Personal Genomics Machine (PGM, Life technologies, USA). Different samples were used for RNA and DNA sequencing.

### Next generation sequencing

#### RNA-sequencing

Total RNA was isolated employing Ultrasense Viral RNA kit (Qiagen, Germany) and concentrated using Ribominus Concentration module (Life technologies, USA). Ribosomal RNA was depleted using Ribominus Eukaryotic kit v2 (Life technologies, USA). Purified mRNA was fragmented using NEBNext^®^ Magnesium RNA Fragmentation Module (New England Biolabs, USA). After adaptor ligation of a library of 200 bp size, cDNA was synthesized and amplified using Ion total RNA sequencing kit v2 (Life technologies, USA) as per manufacturer’s instructions. The cDNA library was quantified and size distribution analysed on High sensitivity DNA chip kit on Agilent Bioanalyzer 2100. Emulsion PCR was carried out using the Ion OneTouch™ 200 Template Kit v2 (Life Technologies, USA) according to the manufacturer’s instructions. Sequencing of the cDNA libraries were carried out on 316/318 chips using the Ion Torrent PGM system and employing the Ion Sequencing 200 kit (Life Technologies, USA) according to the supplier’s instructions. After sequencing, low quality reads and polyclonal sequences were filtered by the PGM software. Sequences matching the PGM 3′ adaptor were also automatically trimmed.

#### DNA sequencing

DNA was isolated from the CSF/serum/plasma samples using QIAamp DNA Mini and Blood Mini Kit (Qiagen, Germany) and from tissue samples by Allprep DNA/RNA mini kit (Qiagen, Germany), and was quantified on Nanodrop 1000 spectrophotometer (Thermo Scientific, USA). DNA was fragmented using Ion Shear Plus Reagents Kit (Life technologies, USA) and purified using Agencourt Ampure XP DNA reagent. The fragmented DNA was ligated with Barcode adapters. Adaptor-DNA constructs were purified, size selected, and amplified via PCR as per the manufacturer’s instructions. Quantification and size distribution analysis was carried out on High sensitivity DNA chip kit on Agilent Bioanalyzer 2100. Emulsion PCR and sequencing of the DNA libraries were carried out on 316/318 chips as per the manufacturer’s instructions.

#### Bioinformatics analysis

Sequencing data size varied from 157 Mb to 2.5 Gb. After trimming quality value (q10) and length (l10), host filtering, 85–95% reads were discarded. Contigs were generated using CLC De-novo Assembler [26] (using de Bruijn graph path finding) and velvet Assembler [27] using optimized K-mer value (velvet optimizer program). The contigs were subjected to BLAST (www.ncbi.nlm.nih.gov/BLAST/). When required, raw reads (≥100nt) were mapped to a reference sequence.

#### Real-time PCR for the detection of PARV4 DNA

The presence and quantitation of HPARV DNA in CSF/plasma/serum samples was evaluated using a real time PCR assay. Sequences of the primers and probe used for this assay were; forward primer (Fwd 5′-CTAAGGAAACTGTTGGTGATATTGCT-3′), reverse primer (Rev 5′-GGCTCTCCTGCGGAATAAGC-3′) and Probe 5′-(FAM) TGTTC AACTTTCTCAGGTCCTACCGCCC -3′) [13]. Amplification reactions were performed using TaqMan 2X Universal PCR master mix (Invitrogen, USA) as per manufacturer’s instruction on Applied BioSystems, 7300 platform. A standard curve was generated from using 10-fold serial dilutions of plasmid DNA containing the 103-bp ORF2 product. Plasmid DNA concentration was determined with a NanoDrop ND-1000 spectrophotometer (Thermo Scientific, USA).

## Full genome/partial NS1 sequencing/phylogenetic analysis

Specimens with high viral load i.e. one CSF and two sera (accession numbers KJ541119–21) were used to generate almost full length genomic sequences using 56 primers (Table 5) and Platinum Pfx DNA polymerase (Life Technologies, U.S.A.) for amplification. In addition, partial NS1 region was amplified for 24 specimens. For this, 5′-GCTCATCCTTTGTTAGGGTACCT-3′ (external forward), 5′-T ACAGCTTTAGCTACTTCAAC-3 (external reverse), 5′-GTTGCACAGTTATT TAGCTTG-3′ (internal forward) and 5′-TCATTCTACCTTCTTCCCACCATACT-3′ (internal reverse) primer sets were used with 35 cycles of one minute each of 94°C, 51°/53°C and 72°C for first/nested runs. The PCR products of predicted molecular size were gel eluted (QIAquick gel extraction kit; Qiagen, Hilden, Germany) and sequenced by using BigDye Terminator cycle sequencing Ready Reaction Kit (Applied Biosystems, U.S.A) and an automatic Sequencer (ABI Prism 3100 Genetic Analyzer; Applied Biosystems). All PCR products were sequenced in both directions. Phylogentic analysis was carried out using MEGA software (version 5.2.2) [28]. For the construction of phylogenetic trees (full genomes, partial NS1), the Neighbor-Joining algorithm and the Kimura 2-parameter distance model were utilized. The reliability of the analysis was evaluated by a bootstrap test with 1000 replications.

**Table 5.** Primers used for PARV4 full genome / partial NS1 amplification and sequencing.

Forward primer 5′→3′	Reverse primer 5′→3′
1 ACGAGGCCTCGTCGCATATG 20	265 GAGTGAGTCAGAGCTGCTGA 246
269 TCGTAGAGGTCACCATGGACG 289	939 TCCTGCCATAGTAGG 925
308 TGCTGCAAATTCCTACCGGA 323	999 TGTGGCTATTCCATTTTCAAC 979
888 CGCACAACCACAAGCACCGG 907	1147 CATTCTTTGTGAGGTACC 1130
1114 GCTCATCCTTTGTTAGGGTACCT1136	1253 CACACTCCTCTTGCCCATGC 1234
1174 GTTGCACAGTTATTTAGCTTG 1194	1967 GGCGGCGCGTCTGCTACTGG 1948
1468 GGAGCTCCCGTGAGGTTAGA 1487	2114 TCTGCATCAAACACGTGTTCT 2094
1508 AGGACTACATACCCACCTG 1526	2436 TTGCATTAGCTCATCTAGT 2418
1668 TGAAGAGGAAGTGAGAAGTT 1687	2467 CCGGTCCAACAGGAATTGC 2449
2055 TGAAAGGTATGGAGCTGGGGACAT 2078	2989 CAATGGAATCAGCTGCTTC 2971
2078 TTGAGGCTTTTTGGTCAGAA 2097	3067 GAGGATTACCAGGACCAACAT 3047
2367 GTAAGCAATCATGTCTGCTGC T 2388	3327 CTGAGAAAGTTGAACATCAG 3308
2541 GGCTTTCCAAGGCTTGC 2557	3587 CTATCAGCTAACATAGAAGT 3568
2928 TCCTCCAGATGTCGGAGTCC2947	3682 TAACATCCATGTAAGAATA 3664
2992 GAACCAGACCTTGAGCGGCCT 3012	3790 TGATTACAATAGCAGAGATTGCAAT 3768
3248 TAGTAGTTGGCAATGCTATT 3267	4163 GGATTATGTCTTGCATACA 4145
3298 GGTGATATTGCTGATGTTCAA C 3319	4260 TGGAAGCCTGCCAACATCTT 4241
3463 ATGTCCGTGGAACCAGCTGG 3482	4367 CTATCACTTGTAGCAGGA 4350
3758 GCCTAACTATTGCAATCTCTG 3778	4586 TGTGGAGCAGCCAAGTCACCAT 4565
3764 CTATTGCAATCTCTGCTATTGT 3785	4658 GGACTGTACACTGTATCAG 4640
4000 GTGGCCATCAGTGATCATA 4018	4997 AATGGAGCAGGTTTCTCGG 4979
4329 GGATCCTGTTGCTATTGG 4346	5059 TGCTTGGGTCTTGTAAGCTAT 5039
4529 GCAGAGTTAGTAATCCATCTAGAGT 4553	5111 TGCGAGTAATTACGCGCAAT 5092
4558 CAGATAGATGGTGACTTGG 4576	5167 CTATAAGAATCAGTCTCACAG C5146
4814 CTCCTCCACCGATGATATTTGT 4834	5183 ACCACACCTACCTCGCCTATAAGAA 5159
4862 CCAGTCCGGGAGCCCATACAGTT 4884	520 GCTTCACTCTGCATGGCAGA 501
4897 TTAAACCAGTATGCC 4911	5259 ATTTCGCTTCCGGTCCCGCG 5244
5061 GCCGGTATATGAAGTTCCTTCTG 5083	5268 GCGTATTTCCGCTTCCGGTCCCGC 5245
**Primers for partial NS1 amplification**	
5′-GCTCATCCTTTGTTAGGGTACCT-3′ (external forward)	5′-T ACAGCTTTAGCTACTTCAAC-3 (external reverse)
5′-GTTGCACAGTTATTTAGCTTG-3′ (internal forward)	5′-TCATTCTACCTTCTTCCCACCATACT-3′ (internal reverse)

### Testing for IgM and IgG antibodies against Orientia tsutsugamushi

In view of the high positivity of these antibodies among patients from subsequent years [8–11], ELISAs (The Scrub Typhus Detect IgM and IgG ELISAs, InBios international, Inc, Seattle, USA) were used to screen that available samples according to the manufacturer’s instructions. These included serum samples from patients and controls (*n* = 92 each). CSF samples were not available. Cut off value of 0.5 was used to discriminate reactive and nonreactive specimens [29]

### Statistical analysis

Chi square test was performed for group comparisons. A *p* value < .05 was considered significant.
